# Survival time and influencing factors among people living with HIV in Guilin City, Guangxi, China: a retrospective cohort study (1996–2022)

**DOI:** 10.3389/fpubh.2025.1575990

**Published:** 2026-01-06

**Authors:** Yan Feng, Liyuan Ge, Wenhui Cheng, Ying Zhuo, Sijia Cao, Jie Tang, Wuxiang Shi, Lingmi Zhou

**Affiliations:** 1 School of Public Health, Guilin Medical University, Guilin, Guangxi, China; 2 School of Humanities and Management, Guilin Medical University, Guilin, Guangxi, China; 3 Guilin Center for Disease Control and Prevention, Guilin, Guangxi, China; 4 The Guangxi Key Laboratory of Environmental Exposomics and Entire Lifecycle Heath Research, Guilin, Guangxi, China

**Keywords:** HIV/AIDS, survival analysis, Kaplan–Meier method, cox proportional hazards regression model analysis, life table method

## Abstract

**Background:**

Despite significant advances in antiretroviral therapy (ART), substantial disparities in survival outcomes among people living with HIV (PLWH) persist in resource-limited settings. However, in Guangxi, a region heavily affected by the HIV/AIDS epidemic in China, research on the survival time of PLWH and its associated factors remains limited. This study aims to investigate the survival time and its influencing factors among PLWH in Guilin, Guangxi, from 1996 to 2022, filling an important gap in regional HIV epidemiological research.

**Methods:**

A retrospective cohort study method was used to study PLWH whose current address was reported as local in Guilin from 1996 to 2022. The life table method and Kaplan–Meier method were used to calculate the survival rate and draw the survival curve, and the Cox proportional hazards regression model was used to analyze the influencing factors of the survival time of PLWH.

**Results:**

A total of 16,068 HIV/AIDS patients were included in the study, with a mortality rate of 8.67/100 person-years. The median survival time of PLWH was 14.11 years (95% CI: 13.50–15.70), and the 1-year, 3-year, 5-year, and 10-year cumulative survival rates were 72, 66, 62, and 54%, respectively. Cox proportional hazards model analysis identified factors significantly associated with increased risk of death, including male sex (HR = 1.425, 95% CI: 1.334–1.522), older age, low education level, unmarried status, farming occupation, and not receiving ART (HR = 10.578, 95% CI: 9.880–11.326). In contrast, individuals infected through heterosexual transmission and those identified through counseling and testing services had better survival outcomes. Lower CD4+T lymphocyte count at enrollment was associated with a higher risk of death.

**Conclusion:**

Survival time of PLWH is affected by many factors. In the context of the continuous improvement of policies and measures of Guilin City’s anti-HIV attack project, behavioral interventions for key populations as well as publicity in rural areas should be strengthened, and early detection and treatment should be advocated in order to improve the quality of survival of PLWH and prolong their survival time. The findings of this study can provide scientific evidence for optimizing HIV prevention and control strategies in similar settings.

## 1 Introduction

Globally, HIV/AIDS, a chronic infectious disease caused by human immunodeficiency virus (HIV), continues to pose a major public health threat (1, 2). According to the latest report of the World Health Organisation (WHO), millions of people are still newly infected with HIV every year, and a large number of infected people fail to receive timely diagnosis and effective treatment, resulting in deterioration of their condition and eventual development of HIV/AIDS (3). This situation not only poses a serious threat to the lives and health of individuals, but also has a far-reaching impact on social equality, economic development, education and social stability around the world.

The spread of HIV/AIDS and the survival of People Living with HIV (PLWH) are affected by a combination of complex factors. Biologically, HIV is characterised by a long latency period and high variability, allowing infected individuals to transmit the virus during the asymptomatic phase, while the destruction of CD4+T lymphocytes by the virus directly weakens the body’s immunity, increasing the risk of opportunistic infections and death. In addition, early detection of infection status and the ability to receive timely antiviral therapy (Whether or not treated with ART) significantly affects disease progression and prognosis.

At the behavioral level, high-risk sexual behaviors, injecting drug use, multiple sexual partners and weak awareness of safety precautions are important drivers of HIV transmission. After diagnosis, patients’ adherence to medication, regular follow-up and adoption of healthy lifestyles also largely determine the quality of their survival and life expectancy.

Socio-economic conditions are likewise one of the key factors influencing the effectiveness of HIV/AIDS prevention and control. Guilin, as an important city in China’s Guangxi Zhuang Autonomous Region, has shown a continuous growth trend in the epidemic, despite the achievements made in HIV/AIDS prevention and control in recent years (4, 5). As of 2022, the cumulative number of reported HIV/AIDS cases in the city has exceeded 10,000, reflecting that the situation of prevention and control in the region is still severe (6). Especially in some remote rural areas and low-income groups, the uneven distribution of medical resources, heavy economic burden, and insufficient health knowledge have limited the effective control of HIV/AIDS.

At the same time, the lack of psychological and social support systems further exacerbates the plight of patients. Many PLWH face severe social discrimination and stigma, leading to frequent mental health problems such as depression, anxiety and even suicidal tendencies. Strained or broken family relationships also leave patients without emotional support, which in turn affects their treatment adherence and quality of life.

At the institutional level, although Guilin has gradually established a prevention and control system that includes voluntary counseling and testing (VCT) clinics, expanding screening coverage, and promoting free antiretroviral treatment since the first case of HIV/AIDS was reported in 1996 (6), the shortage of grass-roots professionals, insufficient publicity, and poor accessibility of services still exist in the process of policy implementation, which constrains the actual effectiveness of prevention and control measures.

Therefore, an in-depth analysis of the survival status of PLWH in Guilin, including their survival rate, survival time and their main influencing factors, is of great significance to optimise the existing intervention strategies, improve the quality of life of the patients and curb the spread of the epidemic. Based on the epidemiological data of PLWH in Guilin between 1996 and 2022, this study will systematically explore the development trend of the epidemic and the effectiveness of prevention and control, with the aim of providing a scientific basis and practical reference for the prevention and treatment of HIV/AIDS at the local and national levels.

## 2 Methods and materials

This study was reported in accordance with the Strengthening the Reporting of Observational Studies in Epidemiology (STROBE) statement (7).

### 2.1 Conceptual framework

**Figure 1:**
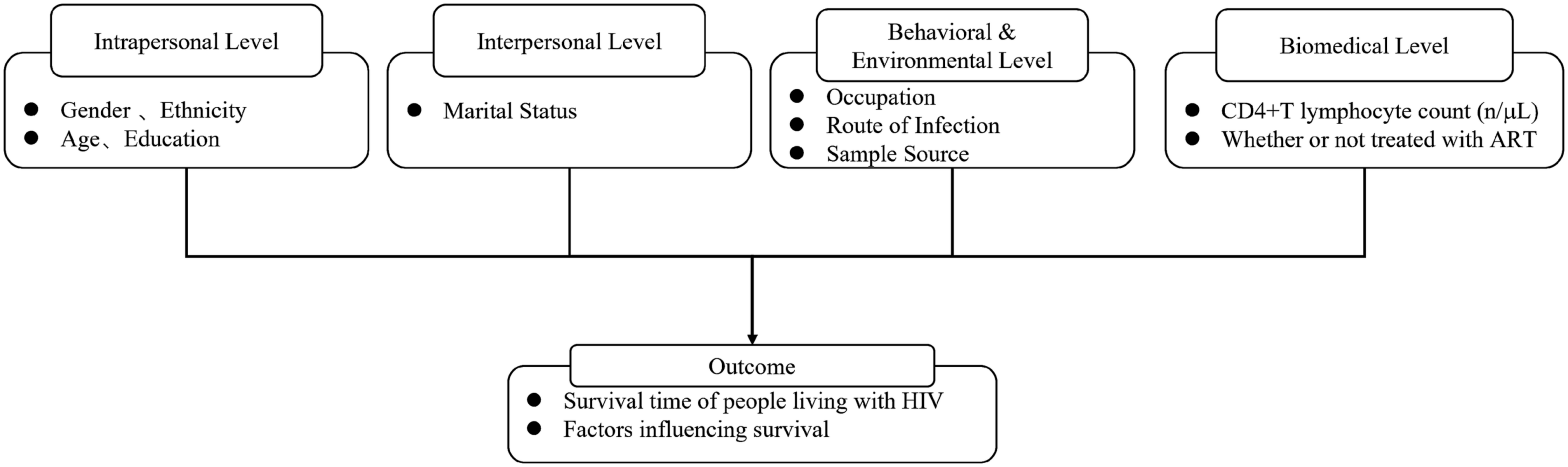
Conceptual framework for factors influencing the survival time of PLWH in Guilin.

An individual’s health outcome is not determined by a single factor, but rather by the interplay of multiple factors across individual, social, environmental, and policy levels (8). This study categorizes factors influencing the survival time of PLWH into four parallel yet interrelated levels: At the individual level, this refers to personal biological, psychological, and demographic characteristics, including variables such as gender, age, ethnicity, education level, and marital status. At the interpersonal level, this involves the individual’s family, friends, and social networks. Although the current dataset does not directly include information on family support, marital status serves as a partial proxy for this level. At the behavioral and environmental level, this encompasses health-related behaviors and the physical and social environments in which individuals live. Specific factors include occupation (reflecting socioeconomic status and work environment), route of infection (indicating high-risk behavior patterns), and sample sources (reflecting health awareness and accessibility of medical services). At the biomedical level, this includes physiological and clinical factors directly related to the disease. Among these, CD4+T lymphocyte count at diagnosis and whether antiretroviral therapy (ART) has been initiated are the most critical factors in this study. This study aims to explore how these four parallel, interrelated levels of factors jointly influence the survival time of people living with HIV in Guilin. Our conceptual framework (Figure 1) illustrates the associations between these factors and the ultimate survival outcomes.

### 2.2 Source of information

This study employed a retrospective cohort study design, with data sourced from the National Comprehensive Information System on HIV/AIDS in China. This system serves as the national platform for reporting notifiable infectious diseases and is characterized by high data quality and completeness. The first HIV/AIDS case in Guilin, Guangxi, was reported in 1996. HIV/AIDS case reports and follow-up data from January 1, 1996, to December 31, 2022, were extracted and downloaded from the National Comprehensive Information System on HIV/AIDS. A total of 16,068 HIV/AIDS patients were included in the study. Survival time for HIV/AIDS patients was calculated from the date of diagnosis; if the diagnosis date was missing, the date of symptom onset was used instead. Inclusion criteria were: (1) current residence in Guilin; (2) confirmed positive result for HIV/AIDS testing; and (3) complete baseline and follow-up information. Exclusion criteria were: (1) cases categorized as foreign nationals or from Hong Kong, Macao, or Taiwan; and (2) cases marked as deleted in the final review. This study is a real-world retrospective cohort study based on the National Comprehensive Information System on HIV/AIDS in China, including all eligible HIV/AIDS cases in Guilin from 1996 to 2022. A whole-population analysis was conducted, and therefore no prior sample size calculation was performed. The sample size was determined naturally by the actual reported data. The study was approved by the Ethics Committee of Guilin Medical College (No. GLMC20240340).

### 2.3 Research method

A retrospective cohort study design was used to collect information on demographic characteristics, routes of infection, sample sources, antiretroviral treatment, and CD4+T lymphocyte counts for the study participants, which were treated as covariates for descriptive analysis. The main variables in this study include: survival time of PLWH, defined as the time from the date of HIV/AIDS diagnosis (or date of symptom onset if the diagnosis date was missing) to the endpoint of observation; the outcome event, which is death; individuals who were lost to follow-up or still alive at the end of the observation period were considered censored data.

All 16,068 eligible PLWH in Guilin from 1996 to 2022 were included in the analytical cohort. For the multivariable Cox regression model, only covariates with zero missing values and strong epidemiological relevance were selected. Variables with any degree of missingness (including those with <20% missing) were not included in the final model to ensure analytical rigor and avoid potential bias from incomplete data. This approach aligns with our pre-specified criterion that covariates must have complete data across all participants to be considered for multivariable modeling.

### 2.4 Statistical methods

SPSS 28.0 was applied to process and analyse the data and R 4.3.1 was used for plotting. Cumulative survival of PLWH was analysed using the life table method, overall survival of patients was estimated using the Kaplan–Meier method, and median and mean survival times were calculated and their 95% confidence intervals (95% CI) were reported. Log-rank tests were used to compare survival differences between subgroups. For multivariable analyses, independent risk factors affecting patients’ survival time were assessed using the Cox proportional hazards regression model, and effect sizes were expressed as risk ratios (HR) and their 95% CI. The test level was *α* = 0.05.

In constructing the Cox proportional hazards regression model, we used stepwise regression and screened for potentially influential covariates in conjunction with previous literature and clinical experience. Criteria for variable inclusion included (1) a clear biological or epidemiological basis, (2) data completeness above 80%, and (3) a statistical association with survival time (*p* < 0.05 on univariate analysis). Variables excluded due to >20% missingness included: antiretroviral therapy (ART) adherence, body mass index (BMI), detailed socioeconomic indicators (e.g., household income, insurance type), and psychosocial or family support measures. These variables were not systematically recorded in the National Comprehensive Information System on HIV/AIDS, particularly during the early study period. Although the survival of HIV patients may be influenced by factors at multiple levels, including individual, family, community, and healthcare institution, the analysis focused on the major prognostic factors at the individual patient level because the data in this study were mainly derived from case reports and did not contain complete multilevel information. In the future, when multicentre or structured survey data are available, it is recommended that multilevel models be further explored to more comprehensively assess the mechanisms of influence. Consequently, the multivariable Cox model was fitted using all 16,068 participants, as all included covariates were fully observed with no missing values. No imputation, case exclusion, or partial-case analysis was required.

## 3 Results

### 3.1 Baseline characteristics of the study population

Among the 16,068 PLWH, the Age of ≥50 years old accounted for 54.80% (8,805/16068); Male accounted for 71.58% (11,501/16068); 70.47% of the occupations were predominantly Farmers (11,323/16068); the Ethnicity was Han (85.66%) (13,764/16068); the education level was high school and below 93.95% (15,096/16068); and 55.19% (8,868/16068) were married with a spouse. The route of infection was Heterosexual transmission (89.66%) (14,407/16068), followed by Homosexual transmission (4.95%) (796/16068) and Drug use (2.44%) (392/16068). The main source of patients was Consultation Testing in 51.51% (8,276/16068), followed by testing by Tests for other patients in 18.96% (3,046/16068). As of December 2022, 94.90% (9,500/10011) of surviving patients in Guilin had received antiretroviral therapy (ART) and were on treatment. The proportion of deceased patients who had received ART was 32.11% (1945/6057).

### 3.2 Survival of PLWH

**Figure 2:**
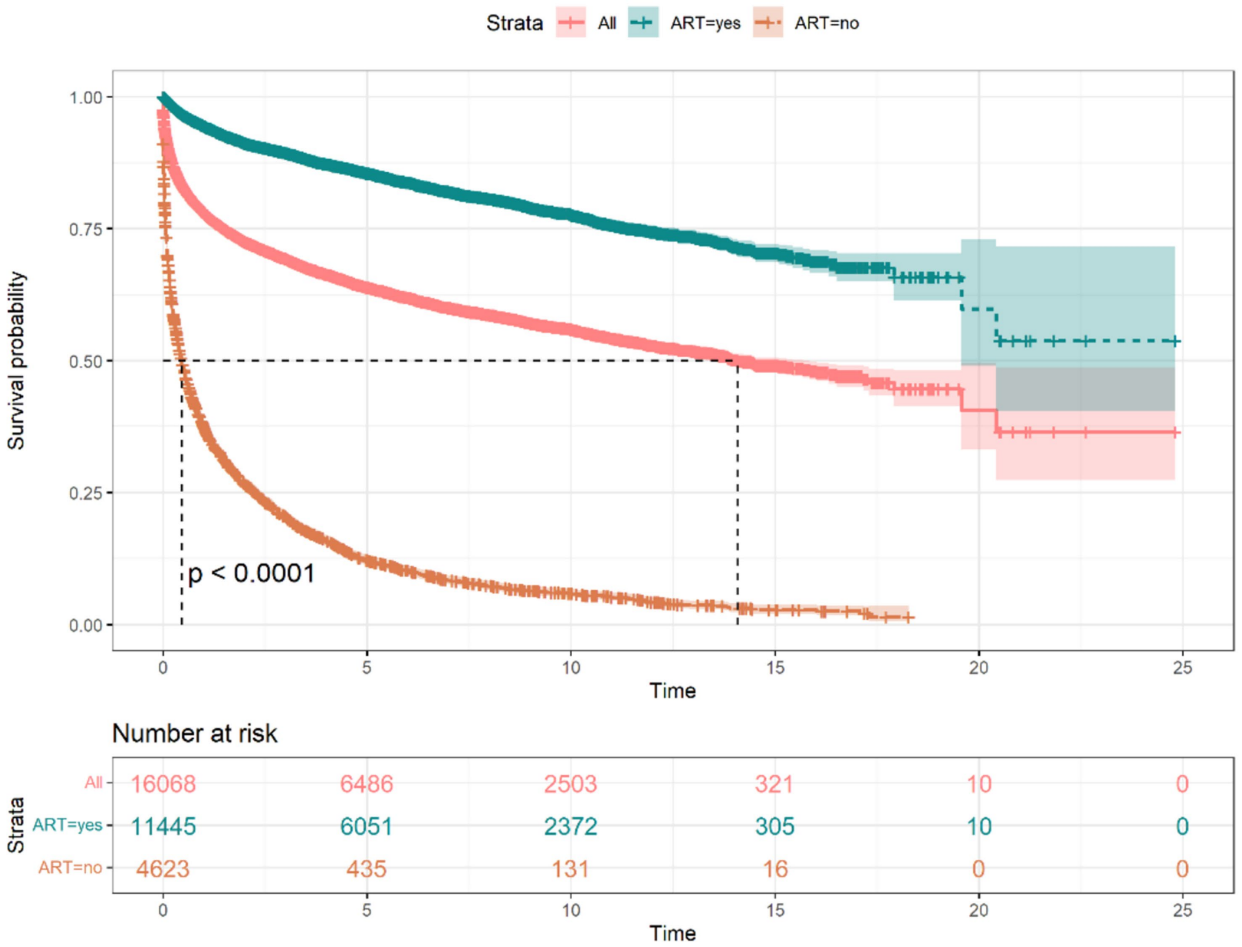
Survival curves of PLWH treated with ART overall and not treated with ART in Guilin.

**Figure 3:**
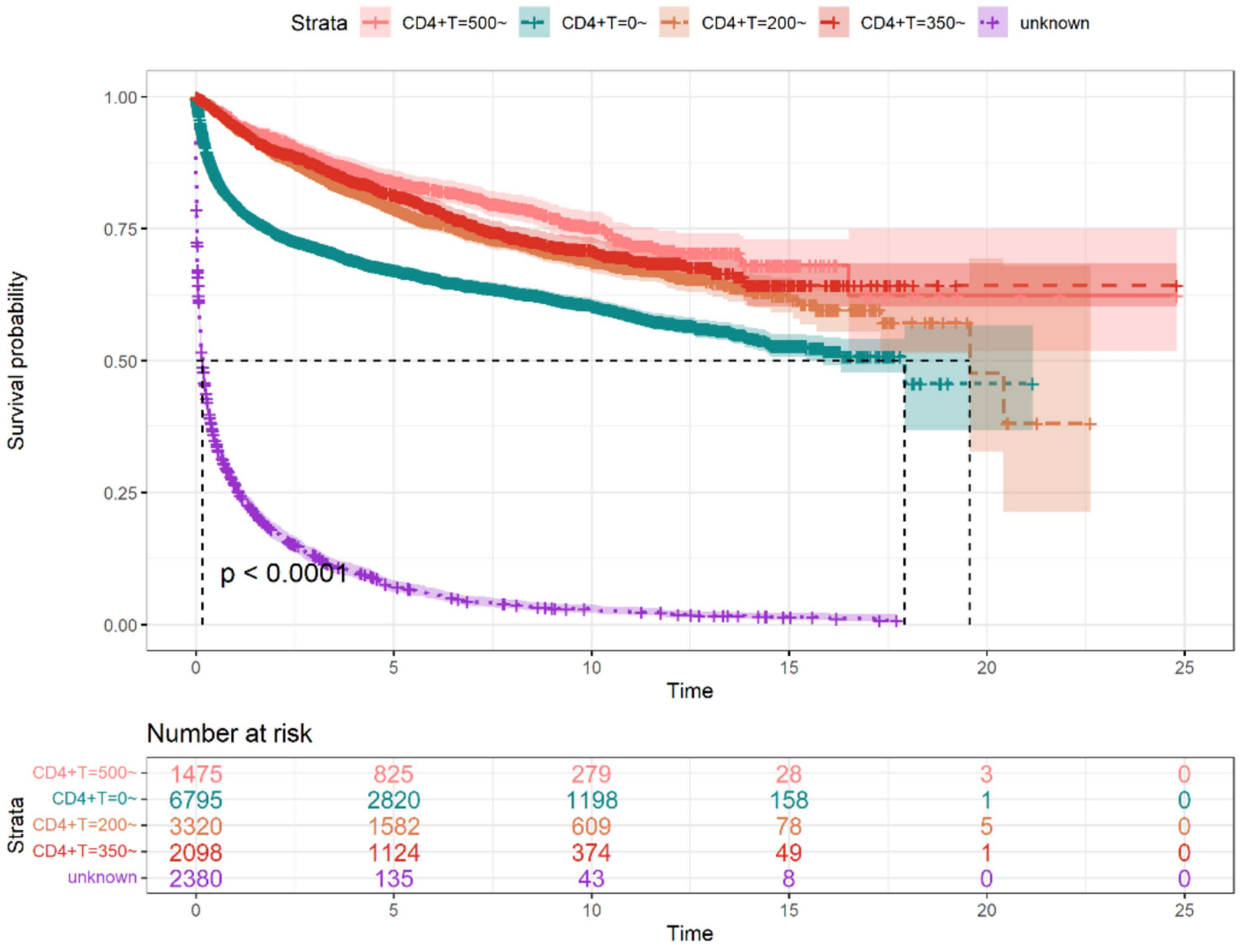
Survival curves of PLWH with different CD4+T lymphocyte counts in Guilin.

**Figure 4:**
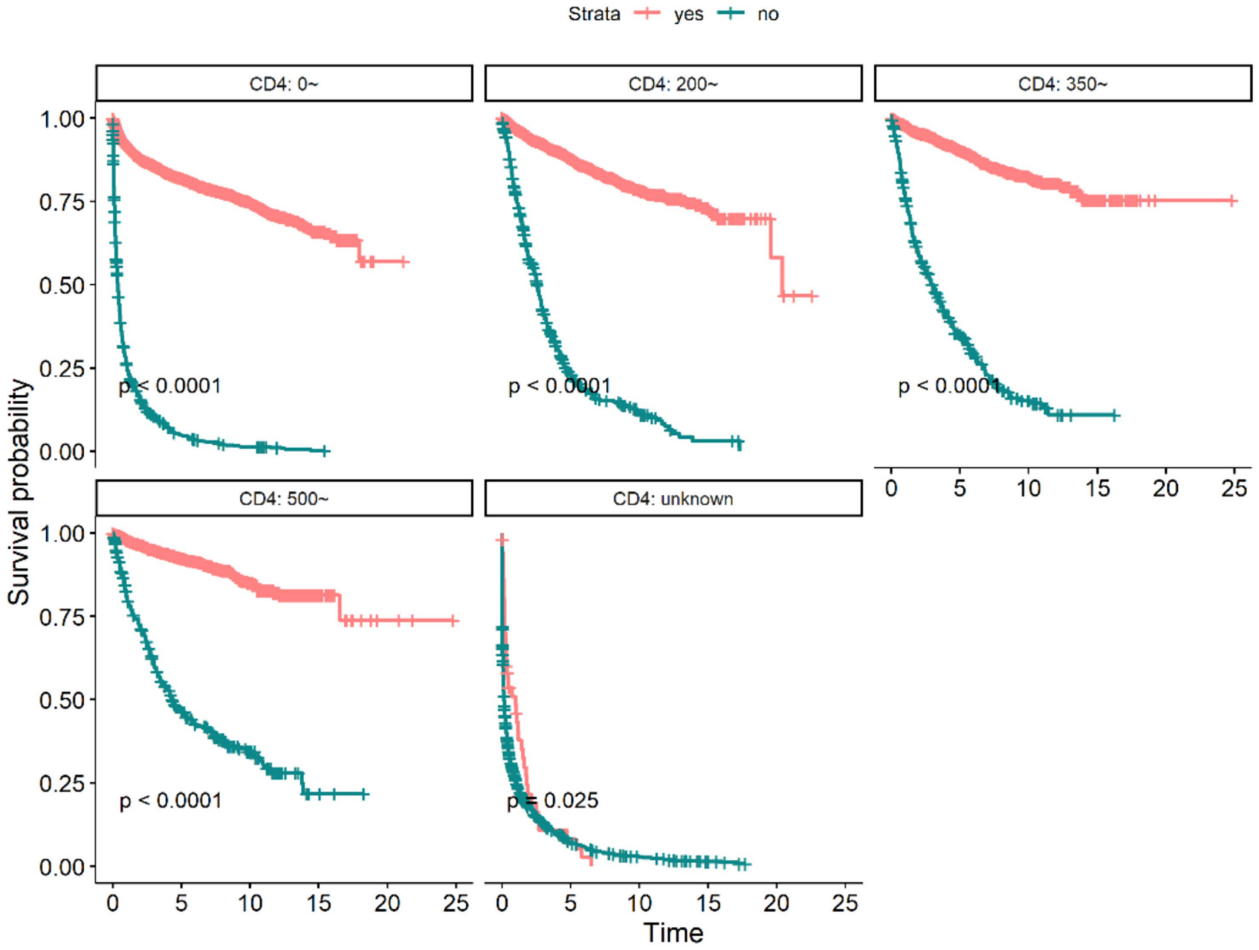
Survival curves of PLWH with different CD4+T lymphocyte counts and different ART treatment status in Guilin.

By the endpoint of observation, 6,057 (37.70%) of the 16,068 PLWH had died, with a follow-up period of 69,859 person-years, with a mean follow-up period of 4.35 years, of which the longest period was 24 years and the shortest period was 0 years, with a mortality rate of 8.67/100 person-years. The 1 year, 3 years, 5 years, and 10 years cumulative survival rates for PLWH were 72, 66, 62, and 54%, respectively, as shown in Table 1. Median survival time has not been observed in PLWH treated with ART; the median survival time in People without HIV treated with ART was 0.460 (95% CI: 0.416–0.507) years (Figure 2). It can also be seen from Figure 2 that the median survival time of overall PLWH in Guilin was 14.11 years (95% CI: 13.50–15.70). The difference in survival rate between study subjects with different values of first CD4+T lymphocyte count assay was statistically significant (χ^2^ = 9837.00, *p* < 0.01), with a median survival time of 17.914 years for those who had a value of <200 on the first CD4+T lymphocyte count assay; 0.162 years for those who had no CD4+T lymphocyte count assay; 19.567 years for those with a CD4+T lymphocyte count assay values of 200–350 had a median survival time of 19.567 years; median survival time has not been observed for those with 350–500; and median survival time has not been observed for those with >500, as shown in Figure 3. And regardless of the starting CD4+T lymphocyte count assay, the median survival time of those who received ART was longer than that of patients who did not receive ART, and the difference was statistically significant (*p* < 0.05) (Figure 4).

**Table 1:** Survival life expectancy of PLWH in Guilin, 1996–2022.

Observation time (years)	Number of initial observers	Deletion	Death toll	Cumulative probability of survival	Standard error of cumulative survival probability
0	16,068	922	3,455	0.78	0.00
1	11,691	927	797	0.72	0.00
2	9,967	822	420	0.69	0.00
3	8,725	854	357	0.66	0.00
4	7,514	761	267	0.64	0.00
5	6,486	880	178	0.62	0.00
6	5,428	667	161	0.60	0.00
7	4,600	665	97	0.59	0.00
8	3,838	602	94	0.57	0.00
9	3,142	582	57	0.56	0.00
10	2,503	519	69	0.54	0.00
11	1915	602	39	0.53	0.00
12	1,274	385	22	0.52	0.00
13	867	313	22	0.50	0.01
14	532	202	9	0.49	0.01
15	321	140	5	0.48	0.01
16	176	82	3	0.47	0.01
17	91	53	3	0.45	0.03
18	35	19	0	0.45	0.00
19	16	5	1	0.42	0.08
20	10	3	1	0.37	0.12
21	6	3	0	0.37	0.00
22	3	1	0	0.37	0.00
23	2	0	0	0.37	0.00
24	2	2	0	0.37	0.00

### 3.3 Analysis of factors influencing survival

#### 3.3.1 Univariate analysis

With the outcome event as the dependent variable, Gender, Age, Ethnicity, Edu-cation, Marital status, Occupation, Route of infection, Sample Source, CD4+T lymphocyte count and Whether or not treated with ART as independent variables, a univariate analysis was conducted. The mean survival time of overall PLWH was 13.288 years (95% CI: 12.713–13.863), and the differences in mean survival time between different groups are shown in Table 2.

**Table 2:** Baseline characteristics and Kaplan–Meier analysis of PLWH in Guilin, 1996–2020.

Factor	Number of observed cases (*n* = 14,062)	Number of deaths (*n* = 5,152)	Mean survival time (years)	95%CI		*χ*^2^	*p*-value
				Lower	Upper		
Gender						356.231	<0.01
Female	4,567	1,227	14.806	14.470	15.143		
Male	11,501	4,830	11.993	11.419	12.568		
Age (years)						933.565	<0.01
15~	187	16	15.176	14.085	16.267		
0~	93	28	12.037	10.523	13.551		
20~	657	107	18.613	17.054	20.172		
25~	6,326	1862	16.194	15.698	16.689		
50~	8,805	4,044	8.856	8.572	9.140		
Ethnicity						184.365	<0.01
Han	13,764	5,252	13.179	12.596	13.763		
Yao	870	301	10.885	10.262	11.507		
Zhuang	834	243	12.567	11.828	13.307		
Other	462	139	10.464	9.744	11.183		
Unknown	138	122	3.979	3.005	4.953		
Education						374.024	<0.01
College and above	816	87	16.212	15.639	16.785		
High school and below	15,096	5,830	13.056	12.485	13.628		
Unknown	156	140	3.953	3.078	4.827		
Marital status						188.027	<0.01
Married	8,868	3,218	14.083	13.242	14.925		
Unmarried	3,482	1,140	13.737	12.575	14.898		
Divorced	3,620	1,631	9.674	8.933	10.415		
Unknown	98	68	8.369	6.491	10.247		
Occupation						217.677	<0.01
Other	3,312	1,027	14.978	13.880	16.076		
Farmer	11,323	4,631	11.703	11.415	11.991		
Housework	1,433	399	13.766	13.099	14.433		
Route of infection						486.070	<0.01
Heterosexual transmission	14,407	5,455	12.241	12.024	12.485		
Homosexual transmission	796	44	14.929	14.236	15.621		
Drug	392	254	10.083	9.055	11.111		
Mother-to-child	79	21	12.669	11.107	14.232		
Sexual contact and injecting drug	47	26	10.881	8.540	13.223		
Blood collection (plasma)	2	2	10.204	7.135	13.273		
Blood transfusion/blood products	4	2	10.418	2.084	18.751		
Unknown	341	253	5.926	5.068	6.785		
Sample sources						929.736	<0.01
Consultation testing	8,276	2,542	12.469	12.225	12.713		
Tests for other patients	3,046	1758	8.345	7.729	8.962		
Pre-operative testing	1,616	718	8.801	8.353	9.249		
Venereal disease clinic	435	193	9.668	8.896	10.440		
Pre-acquired blood (product) testing	655	296	10.272	9.401	11.143		
Other	2040	550	15.763	14.806	16.720		
CD4+T lymphocyte count (n/μL)						9837.276	<0.01
500~	1,475	280	18.259	17.138	19.381		
0~	6,795	2,325	12.720	12.297	13.144		
200~	3,320	763	15.272	14.520	16.024		
350~	2098	461	17.880	17.257	18.503		
Unknown	2,380	2,228	1.297	1.175	1.419		
Whether or not treated with ART						12585.750	<0.01
Yes	11,445	1945	18.259	17.450	19.067		
No	4,623	4,112	2.084	1.963	2.205		

#### 3.3.2 Multivariable cox regression analysis

The multivariable Cox regression model analysis showed that: the risk of death in male was 1.425 times (95% CI: 1.334–1.522) higher than that in female; the risk of death at the age of 25 ~ and 50 ~ was 1.716 times (95% CI: 1.043–2.823) and 2.979 times (95% CI: 1.807–4.913) higher than that at 15~, respectively; education level of high school and below was 1.480 times (95% CI: 1.187–1.846) higher than that of college and above; marital status of unmarried was 1.082 times (95% CI: 1.004–1.166) higher than that of married; farmers’ risk of death was 1.200 times (95% CI: 1.115–1.291) higher than that of occupation other; The risk of death was 0.386 (95% CI: 0.284–0.524) and 0.206 (95% CI: 0.051–0.828) times higher for homosexual transmission and blood transfusion/blood products than for heterosexual transmission, respectively; and the risk of death for Tests for other patients, pre-operative testing, and other was 1.189 times higher than for counseling and testing (95% CI: 1.117–1.266), 0.894 times (95% CI: 0.821–0.972) and 0.664-fold (95% CI: 0.601–0.733), respectively; CD4+T assays with values of 0 ~ n/μL, 200 ~ n/μL, 350 ~ n/μL, and unknown were 2.461 times (95% CI: 2.169–2.793), 1.445 times (95% CI: 1.259–1.659), 1.252 times (95% CI: 1.078–1.453), and 3.576 times (95% CI: 3.144–4.067); the risk of death in patients who did not receive ART was 10.578 times (95% CI: 9.880–11.326) higher than that in patients who did. See Table 3 for details.

**Table 3:** Cox regression model analysis of survival time of PLWH in Guilin, 1996–2022.

Variable	HR	95%CI	*p*-value
Gender			
Female	1		
Male	1.425	1.334–1.522	<0.001
Age (years)			
15~	1		
0~	1.377	0.559–3.392	0.487
20~	1.012	0.597–1.716	0.965
25~	1.716	1.043–2.823	0.034
50~	2.979	1.807–4.913	<0.001
Ethnicity			
Han	1		
Yao	1.167	1.038–1.313	0.010
Zhuang	1.072	0.942–1.220	0.294
Other	1.253	1.058–1.485	0.009
Unknown	0.802	0.494–1.303	0.373
Education			
College and above	1		
High school and below	1.480	1.187–1.846	0.001
Unknown	1.316	0.794–2.183	0.287
Marital status			
Married	1		
Unmarried	1.082	1.004–1.166	0.038
Divorced	1.034	0.973–1.098	0.281
Unknown	0.923	0.708–1.203	0.552
Occupation			
Other	1		
Farmer	1.200	1.115–1.291	<0.001
Housework	0.960	0.850–1.085	0.513
Route of infection			
Heterosexual transmission	1		
Homosexual transmission	0.386	0.284–0.524	<0.001
Drug	0.984	0.850–1.140	0.831
Mother-to-child	1.385	0.582–3.294	0.462
Sexual contact and injecting drug	1.067	0.719–1.583	0.748
Blood collection (plasma)	0.855	0.206–3.554	0.829
Blood transfusion/blood products	0.206	0.051–0.828	0.026
Unknown	1.053	0.901–1.231	0.513
Sample sources			
Consultation testing	1		
Tests for other patients	1.189	1.117–1.266	<0.001
Pre-operative testing	0.894	0.821–0.972	0.009
venereal disease clinic	0.966	0.833–1.120	0.644
Pre-acquired blood (product) testing	1.097	0.970–1.239	0.139
Other	0.664	0.601–0.733	<0.001
CD4+T-lymphocyte count (n/μL)			
500~	1		
0~	2.461	2.169–2.793	<0.001
200~	1.445	1.259–1.659	<0.001
350~	1.252	1.078–1.453	0.003
Unknown	3.576	3.144–4.067	<0.001
Whether or not treated with ART			
Yes	1		
No	10.578	9.880–11.326	<0.001

## 4 Discussion

A total of 16,068 cases of PLWH were detected and reported in Guilin from 1996 to 2022, with a mortality rate of 8.67/100 person-years, and the cumulative survival rates of PLWH at 1 year, 3 years, 5 years, and 10 years were 72, 66, 62, and 54%, respectively. The mortality rate of PLWH in Guilin was higher than that in Chengdu (5.16/100 person-years) (9), Nanchang (2.4/100 person-years) (10), Wuhan (4.22/100 person-years) (11), and Morocco (7.5/100 person-years) (12), etc., and the cumulative survival rates of PLWH at 1, 3, 5, and 10 years were lower than those reported in other regions (13–15). The results show that PLWH in Guilin have a high risk of death, which may be related to insufficient publicity of HIV/AIDS and insufficient coverage of HIV testing.

Multivariable Cox regression analysis showed that the difference in the effect of gender as male, age between 25 and 50 years and greater than 50 years, occupation as farmer, route of infection as homosexual transmission and drug use, sample source as Tests for other patients and other, CD4+T lymphocyte count less than 500 and un-known, and no ART treatment on the death of PLWH was statistically significant, and this result was consistent with the results of the Shanxi Province, Huzhou city study results are consistent (16, 17). In this study, we found that the risk of death of PLWH is lower than that of men, which is consistent with the findings of the literature (18), which may be due to the fact that the multiple roles played by women in the society make them have stronger health awareness, and they are able to better control their condition and reduce the incidence of complications through early detection, timely treatment, and good self-management. The risk of death at the age of diagnosis 50 ~ years is 2.979 times higher than that of 15 ~ years, and the occupation of farmer is 1.200 times higher than that of other occupations, which may be due to the fact that the socioeconomic situation in rural areas may be relatively backward, and the residents’ income level and education are low, which may limit their willingness and ability to invest in their health, and that the older adults PLWH are often accompanied by a variety of chronic diseases, such as cardiovascular disease, diabetes, etc., and these complications can further exacerbate the condition and raise the risk of death (19, 20). The risk of death from Tests for other patients is 1.189 times higher than that from Consultation Testing, which may be due to the fact that the patients themselves suffer from other diseases, coupled with the attack of HIV/AIDS on their own immune system and the decrease in their self-resistance, leading to a higher risk of death, and also indicates that the other attendee testing is an important way of detecting PLWH (21, 22). Since 2010, Guilin has carried out a policy of combating HIV/AIDS, increasing the number of VCT clinics, expanding the scope of HIV/AIDS testing, and screening more PLWH (6), suggesting that the scope of screening will be gradually expanded in the future of HIV/AIDS testing, and advocating “early detection, early treatment.” In addition, through various channels, such as television, radio, Internet, brochures, etc., to popularize the knowledge and harm of HIV/AIDS to the public, and strengthen the awareness of active testing.

The proportion of patients receiving ART among PLWH in Guilin reached 94.90%, much higher than that in the autonomous region or in foreign provinces and cities, such as Liuzhou (72.0%), Liangshan Prefecture in Sichuan (84.3%), Tianshui in Gansu (68.70%), and South Africa (91%) (23–26), which demonstrated that timely acceptance of ART significantly reduced the risk of death in patients with HIV/AIDS. Antiretroviral therapy is currently the most important measure to control the replication of HIV/AIDS virus and promote the reconstruction of immune function (27, 28), the risk of death in patients who did not receive ART is 10.578 times higher than that in patients who received treatment, and it has been demonstrated that antiretroviral therapy can effectively reduce the risk of death associated with PLWH patients (29, 30), and that by taking antiretroviral medication on a regular basis, it can effectively inhibit viral replication, improve the function of the immune system, delay disease progression, and reduce complications, thus improving the survival rate and quality of life of patients (1, 31). Although the rate of antiretroviral treatment for PLWH in Guilin is much higher than in many regions within and outside the autonomous region, demonstrating the remarkable success of the city in HIV/AIDS prevention and control, the overall mortality rate of PLWH in Guilin is higher than in cities such as Chengdu, Nanchang and Wuhan. This phenomenon may be due to multiple and complex factors: first, the physiological differences between individual PLWH, complications, response to treatment, and drug tolerance may lead to different outcomes even when they receive the same treatment; second, although medical resources are sufficient overall, they are unevenly distributed in some remote or economically underdeveloped areas, which affects the continuity and effectiveness of the treatment; and third, the social discrimination faced by PLWH in general and the resulting social discrimination and discrimination. Furthermore, the social discrimination and resulting psychological pressure commonly faced by PLWH not only affects their adherence to treatment, but may also exacerbate the deterioration of their condition; in addition, statistics and comparisons of mortality rates are subject to limitations due to the methods of data collection, processing, and analysis in different regions (32, 33). Therefore, to comprehensively understand and solve the problem of high mortality rate of PLWH in Guilin, it is necessary to comprehensively consider the above factors and take targeted measures to further enhance the treatment effect, improve the quality of life of the patients and reduce the mortality rate.

There are certain limitations in this study. Firstly, due to the long time span of the study, some cases were lost during follow-up, which may introduce a loss-of-visit bias, thus affecting the representativeness and accuracy of the results. Second, due to the limitation of data availability, this study used the time of diagnosis of HIV/AIDS as the starting point of survival analysis, rather than the exact time of HIV infection, which may lead to a certain degree of time bias, affecting the assessment of the real survival status of the patients. In addition, this study was mainly a retrospective analysis based on existing records, and failed to explore in depth the specific efficacy of different antiretroviral treatment regimens and the impact of their side effects on the morbidity and mortality rates, and did not adequately consider the moderating effect of individual differences in patients’ response to treatment. Although our cohort includes all reported PLWH in Guilin, certain prognostically important variables—such as ART adherence, body mass index (BMI), and measures of social or family support—were not included in the multivariable Cox model due to high missingness (>20%) or lack of systematic recording in the national surveillance system. Their exclusion may limit our ability to fully adjust for behavioral and socioeconomic confounders, potentially introducing residual confounding. Finally, although the health status of HIV-infected patients may be influenced by a combination of multilevel factors such as family, community, and healthcare system, multilevel modeling was not used in this study for analysis due to the limitations of the current data structure and types of variables. In terms of statistical analyses, the current multifactor Cox regression model focused on reporting the hazard ratio (HR) of each covariate and its significance level, and has not yet systematically reported the overall fit of the model. Future studies may further explore the effects of multilevel factors on the survival outcomes of PLWH in conjunction with questionnaire surveys or regional health information systems with more complete data support, and introduce methods such as the Likelihood Ratio Test (LRT) in order to assess the statistical significance of the model more comprehensively. In the follow-up work, we will try to construct a model with interaction terms to more comprehensively reveal the combined effects of multiple factors on survival outcomes.

In summary, the survival time of PLWH in Guilin is low and affected by many factors. With the support of the policy of the anti-HIV attack project, it is necessary to continue to expand the coverage of testing and ART, and at the same time to strengthen the publicity in rural areas, advocate early detection and early treatment, so as to effectively reduce the morbidity and mortality rate of PLWH, prolong the survival time, and improve the quality of their lives.

## Data Availability

The original contributions presented in the study are included in the article/Supplementary material, further inquiries can be directed to the corresponding authors.
