# Supplementary material for: Survival time and influencing factors among people living with HIV in Guilin City, Guangxi, China: a retrospective cohort study (1996–2022)
Source: Front Public Health. 2026 Jan 6;13:1575990. doi: 10.3389/fpubh.2025.1575990 (PMC12816266; doi:10.3389/fpubh.2025.1575990)
Supplement: Supplementary file 1 [file Table_1.DOC]

STROBE Statement—Checklist of items that should be included in reports of ***cohort studies***

|  | Item No | Recommendation | Where reported |
| --- | --- | --- | --- |
| **Title and abstract** | 1 | (*a*) Indicate the study’s design with a commonly used term in the title or the abstract | Title: “A Retrospective Cohort Study (1996–2022)” |
|  |  | (*b*) Provide in the abstract an informative and balanced summary of what was done and what was found | Abstract, lines 15–39 |
| Introduction | | |  |
| Background/rationale | 2 | Explain the scientific background and rationale for the investigation being reported | Introduction, lines 40-84 |
| Objectives | 3 | State specific objectives, including any prespecified hypotheses | Abstract, line 18-20; Introduction, last paragraph |
| Methods | | |  |
| Study design | 4 | Present key elements of study design early in the paper | Abstract; Methods and Materials |
| Setting | 5 | Describe the setting, locations, and relevant dates, including periods of recruitment, exposure, follow-up, and data collection | Methods and Materials > Source of information (Guilin, China; 1996–2022; National Comprehensive Information System on HIV/AIDS) |
| Participants | 6 | (*a*) Give the eligibility criteria, and the sources and methods of selection of participants. Describe methods of follow-up | Methods and Materials > Source of information (Inclusion: local residence, confirmed HIV+, complete baseline; Exclusion: foreign/HK-Macao-Taiwan, deleted records; Follow-up until Dec 2022) |
|  |  | (*b*) For matched studies, give matching criteria and number of exposed and unexposed | Not applicable (unmatched cohort) |
| Variables | 7 | Clearly define all outcomes, exposures, predictors, potential confounders, and effect modifiers. Give diagnostic criteria, if applicable | Methods and Materials > Research method (Survival time = diagnosis to death; censoring defined; covariates listed); Table 3 |
| Data sources/ measurement | 8* | For each variable of interest, give sources of data and details of methods of assessment (measurement). Describe comparability of assessment methods if there is more than one group | Methods and Materials > Source of information & Research method (All variables from national case reporting system; ART = ever initiated; CD4 = first test value) |
| Bias | 9 | Describe any efforts to address potential sources of bias | Discussion > Limitations (Loss to follow-up bias, time bias, residual confounding acknowledged) |
| Study size | 10 | Explain how the study size was arrived at | Methods and Materials > Source of information (“Whole-population analysis… no sample size calculation”) |
| Quantitative variables | 11 | Explain how quantitative variables were handled in the analyses. If applicable, describe which groupings were chosen and why | Methods and Materials > Statistical methods; Table 3 (Age, CD4 categorized; categories based on clinical relevance) |
| Statistical methods | 12 | (*a*) Describe all statistical methods, including those used to control for confounding | Methods and Materials > Statistical methods (Cox proportional hazards model with stepwise selection; HR adjusted for listed covariates) |
|  |  | (*b*) Describe any methods used to examine subgroups and interactions | Results > Figure 4 (Stratified survival by CD4 and ART); Discussion mentions future interaction analysis |
|  |  | (*c*) Explain how missing data were addressed | Methods and Materials > Research method  : “All covariates included in the multivariable Cox model had  zero missing values  . Variables with any missingness (e.g., ART adherence, BMI, income) were excluded due to incomplete recording in the national surveillance system.” |
|  |  | (*d*) If applicable, explain how loss to follow-up was addressed | Discussion > Limitations (“Some cases were lost during follow-up… may introduce loss-to-follow-up bias”) |
|  |  | (*e*) Describe any sensitivity analyses | Not performed |
| Results | | |  |
| Participants | 13* | (a) Report numbers of individuals at each stage of study—eg numbers potentially eligible, examined for eligibility, confirmed eligible, included in the study, completing follow-up, and analysed | Methods and Materials > Source of information (N=16,068 included); Results (6,057 died, 10,011 censored) |
|  |  | (b) Give reasons for non-participation at each stage | Methods and Materials > Source of information (Excluded: foreign/HK-Macao-Taiwan, deleted records) |
|  |  | (c) Consider use of a flow diagram | Not included (optional) |
| Descriptive data | 14* | (a) Give characteristics of study participants (eg demographic, clinical, social) and information on exposures and potential confounders | Results > Baseline characteristics; Table 2 |
|  |  | (b) Indicate number of participants with missing data for each variable of interest | Table 2 footnote  (or state here): “  All covariates in the final Cox model had 0% missing values.  Variables not included due to >20% missingness: ART adherence, BMI, household income, insurance type, psychosocial support.” |
|  |  | (c) Summarise follow-up time (eg, average and total amount) | Results > Survival of PLWH (“mean follow-up 4.35 years; total 69,859 person-years”) |
| Outcome data | 15* | Report numbers of outcome events or summary measures over time | Results > Survival of PLWH (“6,057 deaths [37.70%]”) |
| Main results | 16 | (*a*) Give unadjusted estimates and, if applicable, confounder-adjusted estimates and their precision (eg, 95% confidence interval). Make clear which confounders were adjusted for and why they were included | Table 3 (Adjusted HRs); Table 2 (Univariate mean survival) |
|  |  | (*b*) Report category boundaries when continuous variables were categorized | Table 3 (Age groups: 15~, 25~, 50~; CD4 categories: <200, 200–350, etc.) |
|  |  | (*c*) If relevant, consider translating estimates of relative risk into absolute risk for a meaningful time period | Not performed |
| Other analyses | 17 | Report other analyses done—eg analyses of subgroups and interactions, and sensitivity analyses | Figure 4 (Subgroup by CD4 + ART status) |
| Discussion | | |  |
| Key results | 18 | Summarise key results with reference to study objectives | Discussion, first paragraph |
| Limitations | 19 | Discuss limitations of the study, taking into account sources of potential bias or imprecision. Discuss both direction and magnitude of any potential bias | Discussion > Limitations (Loss to follow-up, time bias, residual confounding, lack of multilevel data) |
| Interpretation | 20 | Give a cautious overall interpretation of results considering objectives, limitations, multiplicity of analyses, results from similar studies, and other relevant evidence | Discussion, throughout |
| Generalisability | 21 | Discuss the generalisability (external validity) of the study results | Discussion (Compared with Chengdu, Nanchang, Morocco; notes rural, real-world setting) |
| Other information | | |  |
| Funding | 22 | Give the source of funding and the role of the funders for the present study and, if applicable, for the original study on which the present article is based | Funding section (4 grants listed; no funder involvement in design/analysis) |

*Give information separately for exposed and unexposed groups.

**Note:** An Explanation and Elaboration article discusses each checklist item and gives methodological background and published examples of transparent reporting. The STROBE checklist is best used in conjunction with this article (freely available on the Web sites of PLoS Medicine at http://www.plosmedicine.org/, Annals of Internal Medicine at http://www.annals.org/, and Epidemiology at http://www.epidem.com/). Information on the STROBE Initiative is available at http://www.strobe-statement.org.
